# Morphological Analysis of the Tibial Slope in 720 Adult Knee Joints

**DOI:** 10.3390/diagnostics12061346

**Published:** 2022-05-28

**Authors:** Marc-Pascal Meier, Yara Hochrein, Dominik Saul, Mark-Tilmann Seitz, Friederike Sophie Klockner, Wolfgang Lehmann, Thelonius Hawellek

**Affiliations:** 1 Department for Trauma Surgery, Orthopaedics and Plastic Surgery, University Medical Center Goettingen, Robert-Koch-Straße 40, 37075 Göttingen, Germany; yara.hochrein@stud.uni-goettingen.de (Y.H.); dominik.saul@med.uni-goettingen.de (D.S.); mark-tilmann.seitz@med.uni-goettingen.de (M.-T.S.); friederike.klockner@med.uni-goettingen.de (F.S.K.); wolfgang.Lehmann@med.uni-goettingen.de (W.L.); thelonius.hawellek@med.uni-goettingen.de (T.H.); 2 Kogod Center on Aging and Division of Endocrinology, Mayo Clinic, Rochester, MN 55905, USA

**Keywords:** tibial slope, MRI, knee morphology, knee surgery, measurement methods

## Abstract

*Background*: The tibial slope (TS) defines the posterior inclination of the tibial plateau (TP). The “individual physiological” TS plays a crucial role in knee-joint stability and should be taken into account in knee-joint surgery. The aim of this study was to analyse the specific morphology of the TS for the medial (med) and lateral (lat) TP in relation to patient characteristics and the measurement method. *Methods:* In this retrospective study, MRI images of knee joints from 720 patients (mean age: 49.9 years [±17.14]) were analysed. The TS was assessed using two established methods according to Hudek (TSH) and Karimi (TSK) for the med and lat TP and gender/side specificity was analysed. *Results:* TSH for the med and lat TP showed significantly (*p* < 0.001) different values compared to TSK (TSK_med_: 2.6° (±3.7), TSH_med_: 4.8° (±3.5); TSK_lat_: 3.0° (±4.0), TSH_lat_: 5.2° (±3.9)). The angles of the lat TP were significantly higher than those of the med TP (TSK: *p* < 0.001; TSH: *p* = 0.002). Females showed a higher med and lat TS compared to males (*p* < 0.001). *Conclusions:* The measurement method has an influence on the values of the TS in knee-joint MRIs. The TS is significantly different for the med and lat TP regardless of the measurement method. There are gender-specific differences for the TS.

## 1. Introduction

The morphology of the knee joint is highly complex [1,2,3,4]. Functional relationships between bone and ligaments are not comprehensively characterized [5,6,7]. In this context, the physiological kinematics of the knee joint is a subject of current scientific studies because they have an enormous impact on functional outcome after knee-joint surgery [5,6]. Especially important in this regard is the tibial slope (TS) [6,8]. The TS defines the posterior inclination angle of the tibial plateau [5,9,10] and has a significant influence on tibial translation and knee-joint stability [7,10,11]. Therefore, the TS is important for knee-joint-preservation as well as knee-joint-replacement surgery [8,12,13].

Surgical treatments such as high tibial osteotomy (HTO) or unicondylar (UKA) and total (TKA) knee arthroplasty can change the TS and have an impact on knee biomechanics [14,15,16]. Recent studies have even shown a relationship between the TS and injuries of the anterior cruciate ligament (ACL) [17,18]. The authors pointed out that a TS of more than 12° is a crucial risk factor for ACL ruptures and failure of ACL reconstruction [13,18]. In general, there is a consensus that the “individual physiological” TS should be surgically reconstructed at the best possible rate [5,12,19,20]. In the case of insufficient reconstruction, a deficit of knee-joint flexion can occur [20,21]. Subsequently, exact preoperative planning is necessary [8,12,22].

In order to achieve this, a physiological reference is required for the preoperative planning [23,24,25]. The current literature indicates a highly variable intra-individual TS [7,22,26]. However, the reference values are based on different imaging procedures and measurement methods [5,26,27]. The measurement of the TS in the lateral radiographs of the knee has been described to be limited, because the determination can only be performed on the basis of a single radiological projection [5,27]. On one hand, no assumption can be made about the differences between the medial (med) and lateral (lat) TS [22,27,28], and on the other hand, the anatomical tibial axis cannot be precisely defined [27,29]. In order to achieve the most accurate and compartmentalised assessment of the TS, cross-sectional images in the form of magnetic resonance imaging (MRI) or computed tomography (CT) are recommended [30,31,32,33,34,35].

In the current literature, there are only a few studies investigating TS in an asymptomatic radiological collective using cross-sectional imaging, analysing possible side-, age- and gender-specific differences [7,36].

In summary, it can be concluded that according to the current state of research, there are not enough studies available to define valid physiological reference values or reference intervals of the TS. Therefore, the aim of this study was to analyse the specific morphology of the TS for the med and lat tibial plateau (TP) in relation to patient characteristics (joint side, age and gender) and the approved measurement methods to Karimi et al. [37] and Hudek et al. [38]. Furthermore, possible differences of the TS between healthy and pathological knee joints should be analysed. This should establish a reference for physiologically measured values of the TS.

## 2. Materials and Methods

### 2.1. Patients

Between 2007 and 2020, a total of n = 5627 patients underwent a knee-joint MRI in the Department for Diagnostic and Interventional Radiology of University Medical Center Goettingen. These MRIs were reviewed retrospectively. After applying inclusion and exclusion criteria, 720 patients were included in the final analysis. The study collective was divided into six age groups (21–30, 31–40, 41–50, 51–60, 61–70 and >70 years). In order to achieve a homogeneous age distribution, n = 120 patients were examined in each group in an unfiltered ascending order. The study was approved by the local ethics committee (IRB number 35/7/20) and performed in accordance with the principles expressed in the Declaration of Helsinki. Without exception, the evaluated MRIs were taken as part of routine diagnostics because of clinical symptoms, such as knee pain of unknown origin or in case of suspicion of traumatic injuries. All MRIs were assessed by a senior radiologist and YH, MPM and TH to exclude structural injuries or heavy joint degeneration.

### 2.2. Inclusion Criteria

All examinations accessible via PACS system (Picture Archiving and Communication System) between 1 January 2007 and 31 December 2020 were initially included in the study. Out of these, all patients older than 20 years were included. All MRI scans were performed on patients to assess knee-joint pathologies. Only patients with a Kellgren/Lawrence score [39] less than 3 were classified as “healthy knee joint” (HKJ). Patients with Kellgren/Lawrence Score of 3 or 4 were classified as “pathological knee joint” (PKJ). All MRIs were examined by the internal radiology department as part of the clinical diagnostic procedure. Every report was re-evaluated by MPM, YH and TH in a blinded fashion.

### 2.3. Exclusion Criteria

All patients with fractures, dysplasia or tumours were excluded. Patients who had undergone osteosynthesis or endoprosthesis were likewise excluded. Similarly, the data did not include patients who had any other implants after knee-joint-preservation surgery. Patients with injuries of the cruciate ligaments, collateral ligaments, quadriceps tendon, patellar tendon or with a state after patellar luxation were also excluded. Low-quality MRIs (based on only a few gates), were excluded. In addition, all MRIs with imaging artefacts were ruled out.

### 2.4. MRI Analysis, Parameters and Methods of Measurement

**Figure 1:**
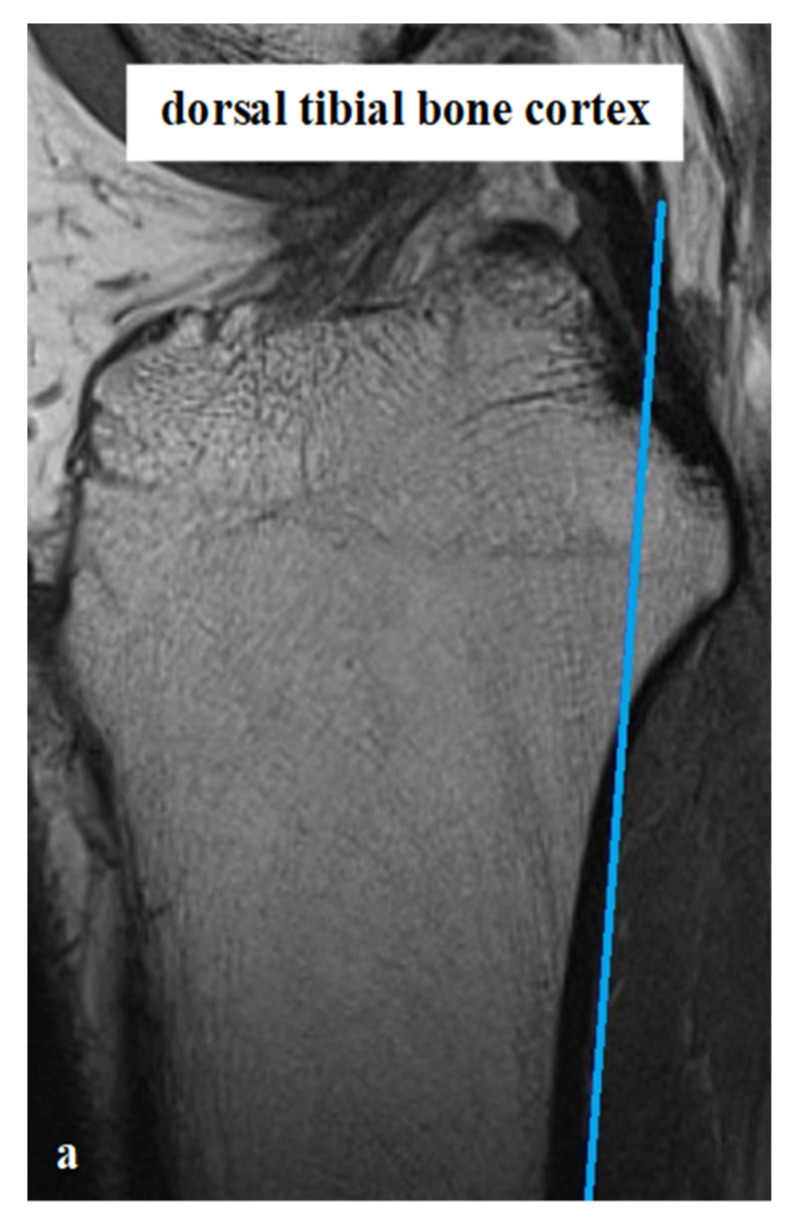

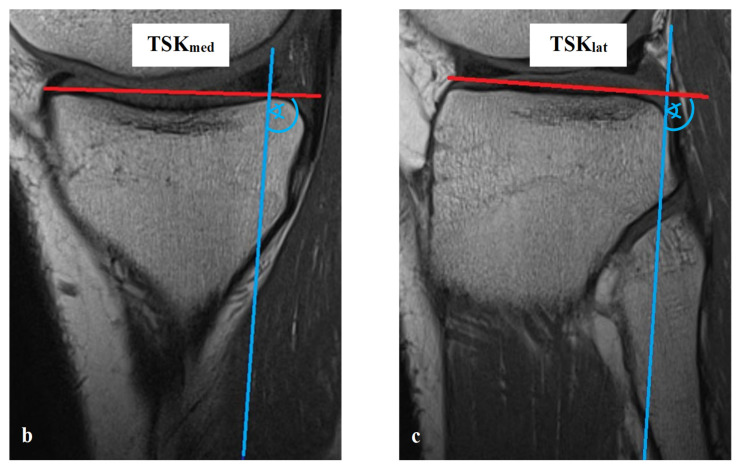
Exemplary depiction of the measurements of the tibial slope according to Karimi et al. [37] for medial (TSK_med_, (**b**)) and lateral (TSK_lat_, (**c**)) knee-joint surface: The measurements of TSK were performed in sagittal view of the knee joint in MRIs. To determine the right position of the dorsal tibial bone cortex for each TSK_med_ and TSK_lat_, a reference line was set in the best available image (**a**). Based on this reference line, the dorsal inclination angle of the tibial plateau was determined in the medial (TSK_med_, (**b**)) and lateral (TSK_lat_, (**c**)) joint sections.

**Figure 2:**
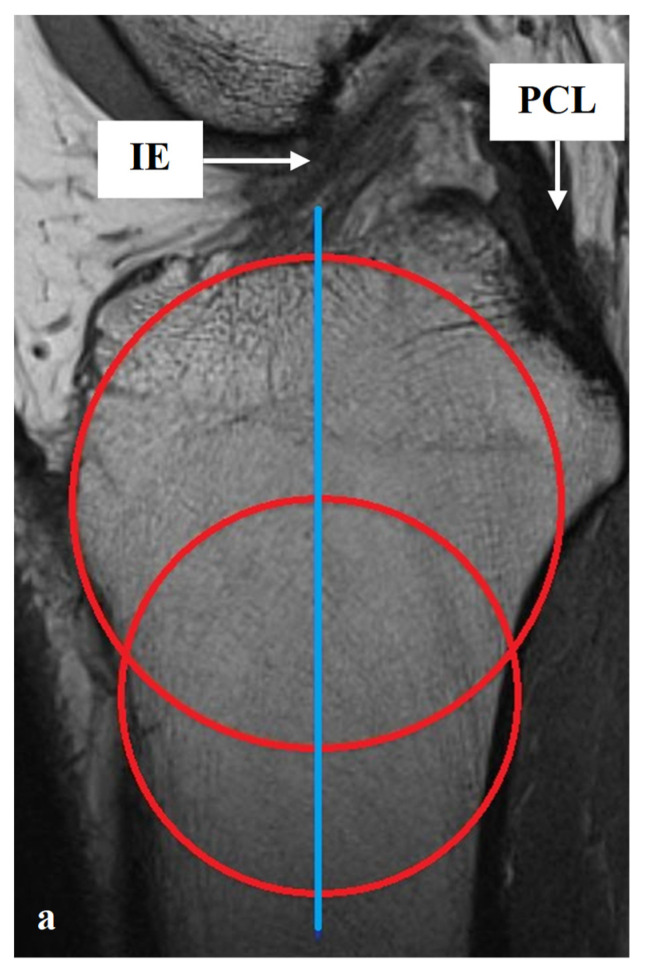

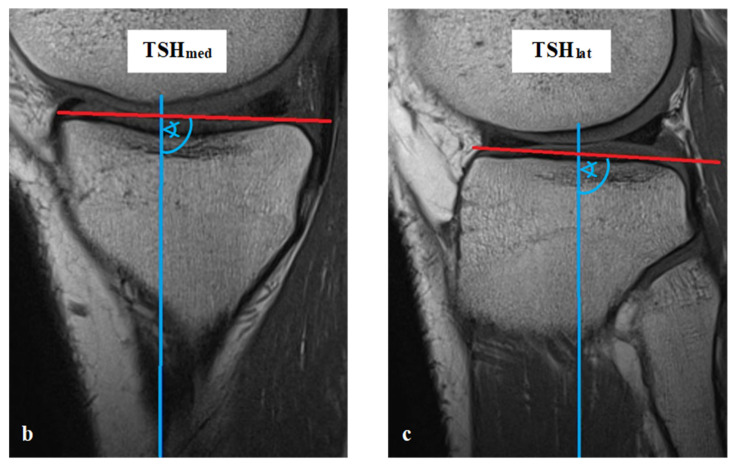
Exemplary depiction of the measurements of the tibial slope according to Hudek et al. [38] for medial (TSH_med_, (**b**)) and lateral (TSH_lat_, (**c**)) knee-joint surface: The measurements of TSH were performed in sagittal view of the knee joint in MRIs. To determine the right position of tibial-shaft axis for each TSH_med_ and TSH_lat_, a reference line was set in the best available image. To detect the tibial-shaft axis as accurate as possible, two virtual circle stencils were used (**a**). The reference structures were the posterior cruciate ligament (PCL) and the intercondyloid eminence (IE). Based on this reference line, the inclination angle of the tibial plateau was determined in the medial (TSH_med_, (**b**)) and lateral (TSH_lat_, (**c**)) joint sections.

All measurements were taken via the PACS system (Picture Archiving and Communication System). Software from GE Healthcare called Centricity^TM^ Universal Viewer was used (RA1000, edition 2019, Buckinghamshire, Great Britain). The osteoarthritis score of each knee joint was classified according to Kellgren/Lawrence (KL), in order to group patients as KJH or KJD. The TS was measured on the medial and lateral knee-joint surface according to Karimi et al. (TSK_med_ and TSK_lat_) [37] and Hudek et al. (TSH_med_ and TSH_lat_) [38]. As a reference for determining the inclination of the tibial plateau, Karimi et al. described the dorsal tibial bone cortex and Hudek et al. the tibial-shaft axis. Figure 1 and Figure 2 show the principle of the measurement methodology.

### 2.5. Statistics

For the statistical comparison of TSK and TSH, the Mann–Whitney U test was used. The analysis was performed separately for the medial and lateral articular surfaces between the TSK and TSH. To investigate possible differences between the medial and lateral knee-joint sections, for each TSK and TSH, a paired *t*-test was established. For side- and gender-specific analyses of TSK_med_, TSK_lat_, TSH_med_ and TSH_lat_, the Mann–Whitney U test was used. Likewise, the Mann–Whitney U test was implemented for comparison of HKJ and PKJ for all assessed TS parameters. In order to detect possible inter-relationships between the different TS parameters and patient age, a Spearman correlation was set. Overall, mean ± standard deviation is stated. Statistical analysis was performed with GraphPad Prism 9.00 (GraphPad Software, San Diego, USA), SPSS Statistics software version 27.0 (IBM SPSS Inc., Chicago, IL, USA) and Microsoft Excel (Microsoft Office 2016, Redmond, USA). Significant differences are marked with asterisks (*** *p* < 0.001, ** *p* < 0.01, * *p* < 0.05).

## 3. Results

### 3.1. Characteristics of the Study Population

In this study, 360 males (50%) and 360 females (50%) were analysed. Likewise, the number of left and right knees was equal. The mean age of the study population was 49.9 years (±17.14). All results are summarised in Table 1.

### 3.2. Differences between TSK and TSH

The analysis of medial knee-joint compartment showed a significant difference between TSK and TSH (*p* < 0.001). The mean TSK_med_ was 2.6° (±3.7), while the mean TSH_med_ was 4.8° (±3.5). Likewise, the measured values of TSH on the lateral joint surface were on average larger than that of TSK (TSK_lat_: 3.0° (±4.0); TSH_lat_: 5.2° (±3.9). The difference was significant (*p* < 0.001). All results are summarised in Table 1.

### 3.3. Differences between Medial and Lateral Knee-Joint Surface

The mean medial TSK (2.6° (±3.7)) was significantly (*p* < 0.001) less than the mean lateral TSK (3.0° [±4.0]). Simultaneously, the analysis showed a significant difference between TSH_med_ and TSH_lat_ (*p* = 0.002). The mean TSH_med_ value was 4.8° (±3.5), while the mean TSH_lat_ was 5.2° (±3.9). All results are summarised in Table 2.

### 3.4. Analysis of Gender Specific Differences

Significantly larger TS values were found in female knee joints. The mean TSK_med_ of the included men was 2.0° (±3.9), while women had a mean TSK_med_ of 3.2° (±3.4). The TSK_med_ values differed significantly (*p* < 0.001). In the case of TSK_lat_, a mean of 2.5° (±3.9) was found in male knees and a mean of 3.6° (±3.9) in female knees (*p* < 0.001). Moreover, the TSH_med_ of analysed men (4.0° [±3.4]) was significantly smaller than that of analysed women (5.6° [±3.4], *p* < 0.001). Likewise, for the mean TSH_lat_, a significant (*p* < 0.001) difference was detected between male knee joints (4.4° [±3.8]) and female knee joints (6.0° [±3.8]. All results are summarised in Table 2.

### 3.5. Analysis of Side Specific Differences

No investigation of any TS parameter showed a side-depending significant difference. TSK_med_ was 2.6° (±3.7) for the left as well as the right knee joint (*p* = 0.815). The mean TSK_lat_ was 3.0° (±3.8) in the left knees and 3.0° (±4.1) in the right knees (*p* = 0.920). On both sides, a mean TSH_med_ of 4.8° was measured. There was no significant side difference (*p* = 0.837). In left knee joint, a TSH_lat_ of 5.2° (±3.8) was found, while in right knee joint, a mean TSH_lat_ of 5.1° (±4.0) was detected. The difference was not significant (*p* = 0.335). All results are summarised in Table 2.

### 3.6. Comparison of HKJ and PKJ

No significant difference was found between the TSK_med_ of HKJ and PKJ (*p* = 0.693). In both groups, the mean TSH_med_ was 2.6° (±3.7). The analysis showed a significantly smaller TSK_lat_ in HKJ (2.8° (±3.9)) than in PKJ (3.4° (±4.0), *p* = 0.032). Likewise, as seen in TSK_med_, the analysis of TSH_med_ was not significantly different between the groups (HKJ: 4.8° (±3.5); PKJ: 4.8° (±3.5); *p* = 0.885). The mean TSH_lat_ (4.9° (±3.9)) of HKJ was smaller than of PKJ (5.5° (±3.9), but the difference was not significant (*p* = 0.091). All results are summarised in Table 2.

### 3.7. Age-Dependent Correlation Analysis of TS Parameters

**Figure 3:**
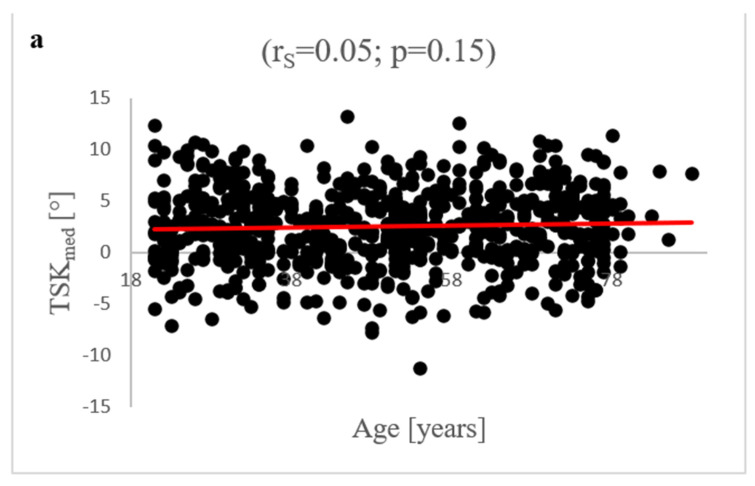

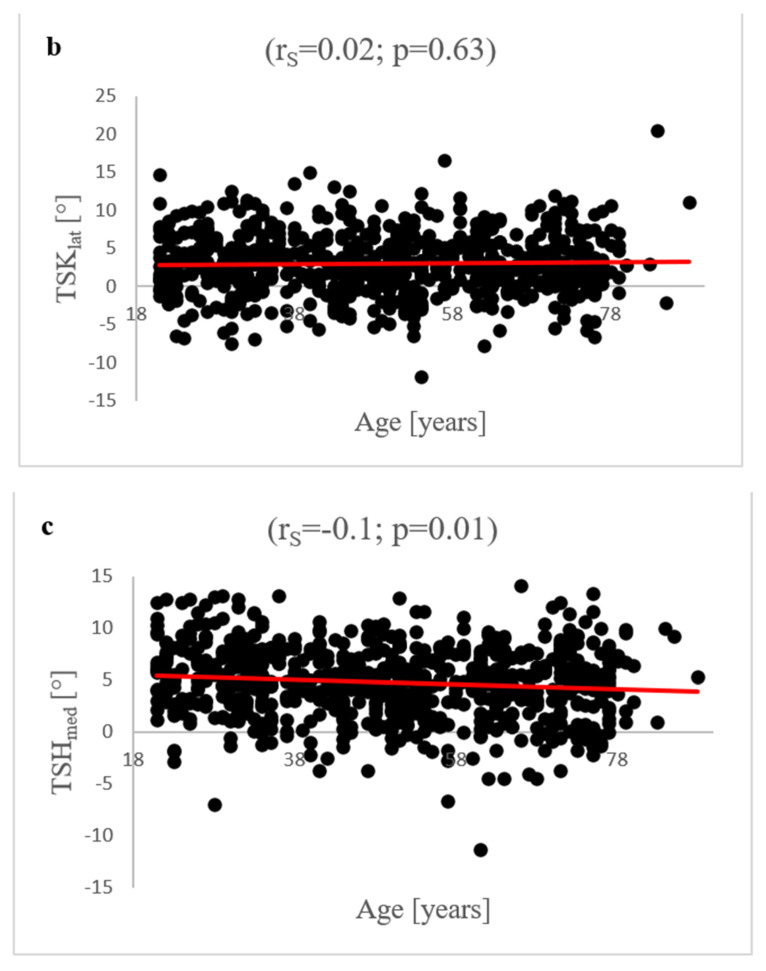

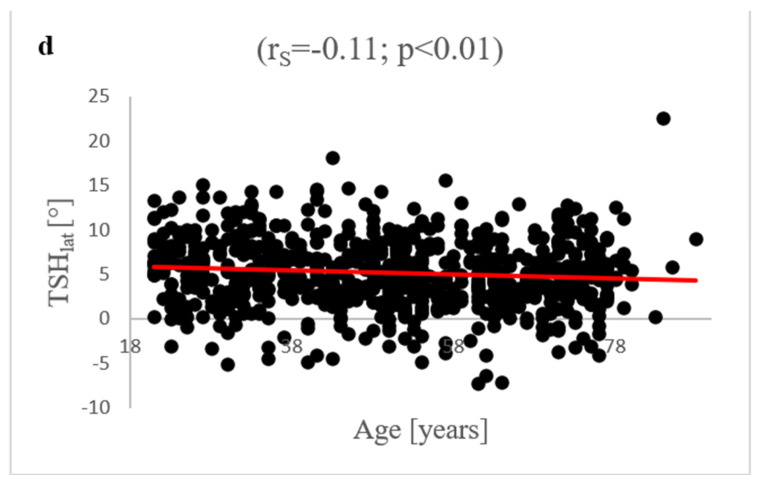
Correlation analysis between patients’ age and TSK_med_, TSK_lat_, TSH_med_ and TSH_lat_: There was no significant Spearman correlation between TSK_med_ (**a**)/TSK_lat_ (**b**) and patient age (TSK_med_: r_S_ = 0.05; *p* = 0.15; TSK_lat_: r_S_ = 0.02; *p* = 0.63). The analysis detected a significant correlation of TSH_med_ (**c**) and TSH_lat_ (**d**) with patient age. A r_S_ of −0.1 (*p* = 0.01) was found in the case of TSH_med_ and a r_S_ of −0.11 (*p* < 0.01) in the case of TSH_lat_.

Between patient age and TSK_med_, no significant correlation was found (r_S_ = 0.05; *p* = 0.15). In addition, no significant correlation was detected for TSK_lat_ and age (r_S_ = 0.02; *p* = 0.63). There was a significant correlation between age and TSH_med_ (r_S_ = −0.1; *p* = 0.01) as well as age and TSH_lat_ (r_S_ = −0.11; *p* < 0.01). The correlation strength according to Spearman was weak. All results are summarised in Figure 3.

## 4. Discussion

The TS is of enormous importance for the biomechanics of the knee joint, especially for tibial translation and general knee-joint stability [7,10,11]. Subsequently, the TS is of major relevance for both knee-joint-preservation and knee-joint-replacement surgical therapy of knee-joint pathologies [8,12,13]. Therefore, it is necessary to radiologically record the TS in the best possible way and to create valid reference values in order to reconstruct the physiological TS in the best manner on the basis of these data [23,24,25]. In order to be able to exactly record both the medial and lateral TS, we examined MRIs in the present study to establish reference values for future therapeutic applicability.

A comparison of the measurement methods according to Karimi et al. and Hudek et al. revealed significantly higher measurement values using the Hudek method in both joint compartments. These results support the current state of research, which shows a large discrepancy between the different measurement methods [7,22,26,40]. Naendrup et al. retrospectively analysed radiological images of 20 patients who underwent revision surgery of the anterior cruciate ligament [40]. As these patients had radiographs as well as CTs and MRIs, a comparison was made between the measurement methods according to Dejour et al. [10] and Utzschneider et al. [22] on the basis of lateral radiographs, as well as Hudek [38] et al. and Hashemi et al. [41] on the basis of MRI and CT examinations. In addition, a comparison was made with the measurement method according to Zhang et al. [31] using 3D CT reconstructions. The authors demonstrated a mean difference of 5.4° for the medial TS and 4.9° for the lateral TS. In comparison to our study, these differences were even larger (difference between TSK and TSH: medial, 2.2°; lateral, 3.2°). In clinical practice, the exact measurement methodology of the TS must be documented, as different measurement methods produce discrepant results and are not comparable with each other.

The measurement of the TS in lateral radiography is limited. Especially, differences between the medial and lateral knee-joint compartment are not exactly assessable a priori [5,27,40]. Our study demonstrated a significantly higher TS on the lateral articular surface, independent of the measurement method that was used. The difference was 0.4° for each TSK and TSH. Jahn et al. retrospectively examined images of 81 patients to compare measurements of medial and lateral TS between radiographs and MRI [42]. The authors pointed out that in an exactly lateral radiograph, the medial and lateral TS can obtain reproducible measurements. Comparable to our results, Jahn et al. found significantly higher measurement values for the lateral TS (medial: 3.7° (±3.3); lateral: 5.7° (±3.7); difference: 2°) in MRI imaging. However, the evaluations of the X-ray images yielded larger values for the medial TS (medial: 8.7° (±3.6); lateral: 7.9° (±3.4)). In contrast, Naendrup et al. found consistently larger measured values for the lateral TS within the scope of the investigated measurement methods based on MRI and CT scans. For example, the evaluations of the 3D reconstructions showed a medial TS of 7.8° (±4.7), compared to a lateral TS of 8.5° (±4.6). In summary, there are differences between the medial and lateral TS. As cross-sectional imaging can be used to exactly determine the lateral and medial knee-joint compartments, this examination method should be attributed to a higher accuracy than the determination on the basis of X-rays. According to the results of the presented study, the physiological lateral TS tends to be larger than the medial TS.

Ni et al. [43] showed in their study, that there is a significant difference in the determination of the TS between scans with and without images of the complete anatomical tibial axis. The authors measured average TS values using the anatomic axis 15.9° (±3.7) and 14.1° (±3.7) at full-length and half-length in true lateral tibial radiographs. Considering these results, a comparison between measurements of the TS in MRI with an incompletely imaged tibial-shaft axis and measurements in CT with a fully imaged axis would be highly interesting.

The results of our study indicate that the TS for the medial articular surface as well as for the lateral surface seems to be higher in female than in male knees, independent of the measurement method. Bao et al. recruited eight healthy volunteers to capture the TS by CT reconstruction [44]. Likewise, they found higher measurement values for the medial and lateral TS in female knee joints, but the results only differed significantly for the medial TS (male: 6.30° (±2.43); female: 7.22° (±2.24)). Contrasting results are provided by the study of Pangaud et al. [7]. The authors reviewed 378 patients with a CT-based modelling and analytics system (SOMA; Stryker). They found a significantly higher TS in male (6.5°, (6.2–6.8)) patients compared to female patients (6.0°, (5.8–6.3)). According to current data, the gender-dependent nature of TS shows an inhomogeneous situation. It can be stated that the TS seems to differ between men and women. The extent to which physiologically higher values are present in one gender compared to the other cannot be conclusively clarified. Using two measurement methods, the present study suggests a stronger dorsal inclination of the tibial plateau in female knee joints.

According to our results, there do not seem to be side-dependent differences in the TS. Likewise, Bao et al. found no significant difference for left and right knees, independent of joint compartment. It can be concluded from this that regardless of whether in knee-joint-preservation or replacement therapy, the TS of the opposite side can be used as a physiological reference and the reconstruction can be planned preoperatively on the basis of this healthy knee-joint side.

To our knowledge there is no study comparing the TS between patients with and without osteoarthritis of the knee joint. According to the present study, osteoarthritic changes of the TS seem to develop primarily in the lateral articular surface. As only the TSK_lat_ shows significant differences between HKJ and PKJ, no clear relationships between osteoarthritic changes of the knee joint and changes of the TS can be defined.

With regard to an age dependency of the tibial slope, we only find significant correlations for TSH. However, the respective effect size is low. To our knowledge, there is no comparative study in the current literature that investigates an age dependency of TS. Consequently, it must be concluded that there are little to no correlations between patient age and tibial slope.

The results of the present study are of major importance for both joint-preserving and joint-replacing surgical therapy of knee-joint pathologies. In general, there is a consensus that the “individual physiological” TS should be respected and surgically reconstructed at the best possible rate [5,12,19,20]. In the case of insufficient reconstruction, a deficit of knee-joint flexion can occur [20,21]. An increased TS widens the flexion gap, improves the leverage of the quadriceps and reduces the patellar contact pressure. However, it can also weaken the tibial attachment of the posterior cruciate ligament (PCL) [45]. That can result in an increased attrition of the polyethylene inlay in knee-joint arthroplasty [45]. A reduction in the tibial slope can negatively affect the postoperative range of motion. It also leads to stress peaks on the anterior part of the tibial plateau [45]. In addition, a decreased TS seems to be a risk factor for primary injuries of the PCL [46] and a TS of 12° and more is associated with a higher risk for ACL injuries after reconstruction [13].

These aspects indicate that the reconstruction of a physiological TS is very important. The results of the present study should be considered as an important reference for TS reconstruction in a physiological manner in knee-joint-preserving and replacing therapy.

## 5. Limitations

A limitation of the study is that there was no comparison of the MRI scans with the related X-ray imaging because many of the included patients were only examined via MRI and not via X-ray. A comparison between the different modalities seems useful. The present study could thereby have made an even better comparison with the results of other authors.

## 6. Conclusions

In the present study, the MRIs of 720 patients were investigated to examine the TS in the medial and lateral knee-joint compartment. We proved that the respective measurement methodology has a significant influence on the measured values of the TS. In clinical practice, the measurement method used should be precisely defined for better comparability. Furthermore, significant differences between the medial and lateral TS were demonstrated.

For the TS, gender-specific differences were detected. Furthermore, higher values for the TS in female than in male knees were found. Joint side had no influence on the TS. There were significant correlations between age and TSH. However, the respective effect size was low. For the first time osteoarthritic changes were analysed in comparison to the TS. We detected a correlation between degenerative changes and the TS only in the lateral joint compartment. To support this thesis, further studies are necessary. In summary, the results of the present study shed new light on the TS and help to better understand the knee joint. Therefore, these results should be considered in the joint-preserving and replacing surgical therapy of knee-joint pathologies in order to reconstruct a physiological TS in the best possible way.

## Figures and Tables

**Table 1:** Analysis of differences between TSK and TSH as well as between medial and lateral articular surface (*** *p* < 0.001, ** *p* < 0.01).

		TSK (n = 720)	TSH (n = 720)	*p*-Value
**medial**	[°]	2.6 (±3.7)	4.8 (±3.5)	<0.001 ***^1^
**lateral**	[°]	3.0 (±4.0)	5.2 (±3.9)	<0.001 ***^1^
		**medial (n = 720)**	**lateral (n = 720)**	***p*-value**
**TSK**	[°]	2.6 (±3.7)	3.0 (±4.0)	<0.001 ***^2^
**TSH**	[°]	4.8 (±3.5)	5.2 (±3.9)	0.002 **^2^

^1^ Mann–Whitney U test, ^2^ Paired *t*-test.

**Table 2:** Gender- and side-specific analysis of TSK_med_, TSK_lat_, TSH_med_ and TSH_lat_ and comparison between HJK and PKJ (*** *p* < 0.001, * *p* < 0.05).

		Males (n = 360)	Females (n = 360)	*p*-Value
**TSK_med_**	[°]	2.0 (±3.9)	3.2 (±3.4)	<0.001 ***^1^
**TSK_lat_**	[°]	2.5 (±3.9)	3.6 (±3.9)	<0.001 ***^1^
**TSH_med_**	[°]	4.0 (±3.4)	5.6 (±3.4)	<0.001 ***^1^
**TSH_lat_**	[°]	4.4 (±3.8)	6.0 (±3.8)	<0.001 ***^1^
		**left (n = 360)**	**right (n = 360)**	***p*-value**
**TSK_med_**	[°]	2.6 (±3.7)	2.6 (±3.7)	0.815 ^1^
**TSK_lat_**	[°]	3.0 (±3.8)	3.0 (±4.1)	0.920 ^1^
**TSH_med_**	[°]	4.8 (±3.5)	4.8 (±3.4)	0.837 ^1^
**TSH_lat_**	[°]	5.2 (±3.8)	5.1 (±4.0)	0.335 ^1^
		**HKJ (n = 409)**	**PKJ (n = 311)**	***p*-value**
**TSK_med_**	[°]	2.6 (±3.7)	2.6 (±3.7)	0.693 ^1^
**TSK_lat_**	[°]	2.8 (±3.9)	3.4 (±4.0)	0.032 *^1^
**TSH_med_**	[°]	4.8 (±3.5)	4.8 (±3.5)	0.885 ^1^
**TSH_lat_**	[°]	4.9 (±3.9)	5.5 (±3.9)	0.091 ^1^

^1^ Mann–Whitney U test.

## Data Availability

All data generated or analysed during this study are included in this published article.
